# Supplementary figures and images for: A combination of curcumin, vorinostat and silibinin reverses Aβ-induced nerve cell toxicity via activation of AKT-MDM2-p53 pathway
Source: PeerJ. 2019 Apr 26;7:e6716. doi: 10.7717/peerj.6716 (PMC6487801; doi:10.7717/peerj.6716)

## Slide 1
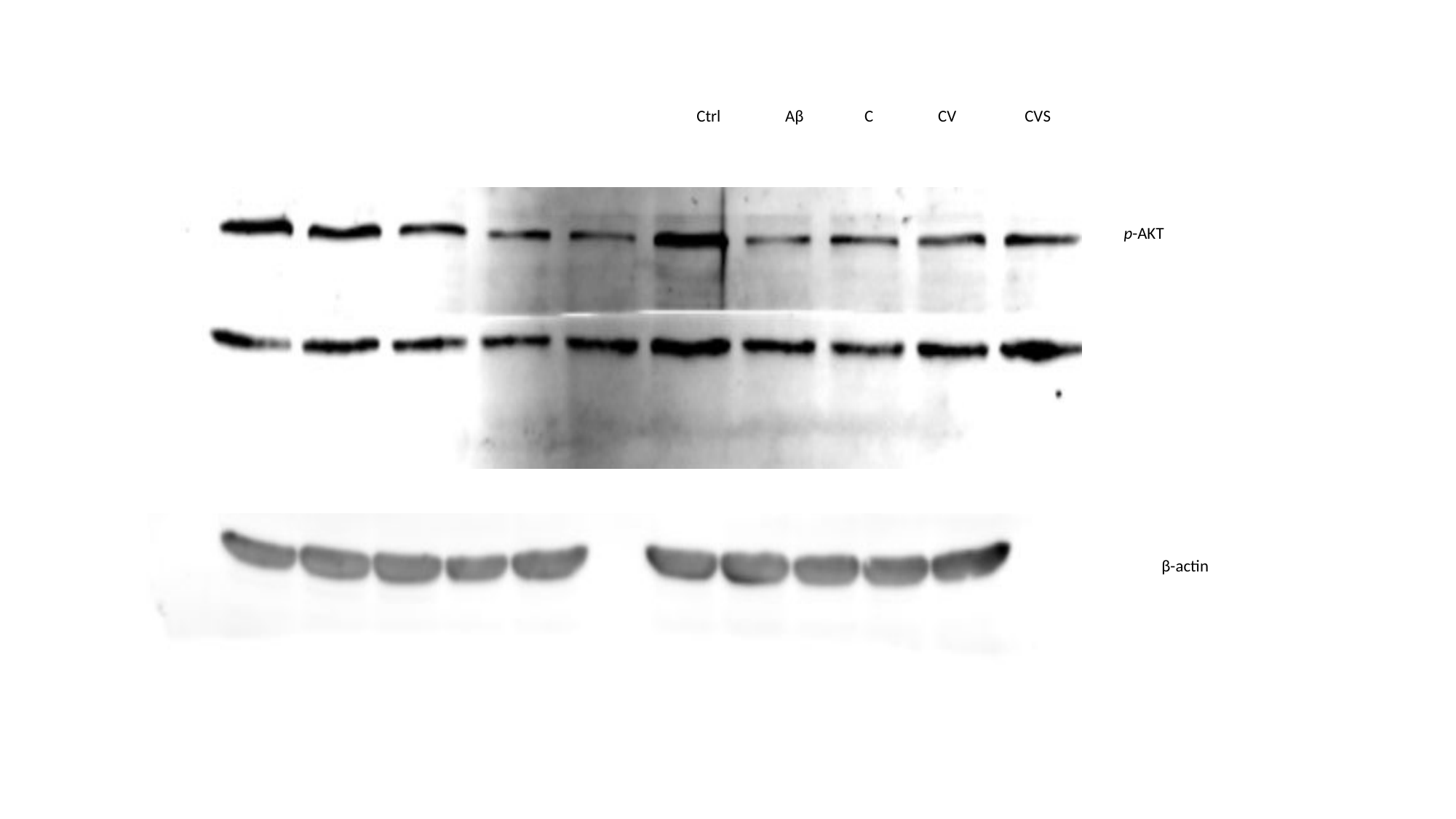

Ctrl Aβ C CV CVS
p-AKT
β-actin

Supplement: Supplemental Information 1 [file peerj-07-6716-s001.zip › Figure 3A.pptx]

## Slide 1
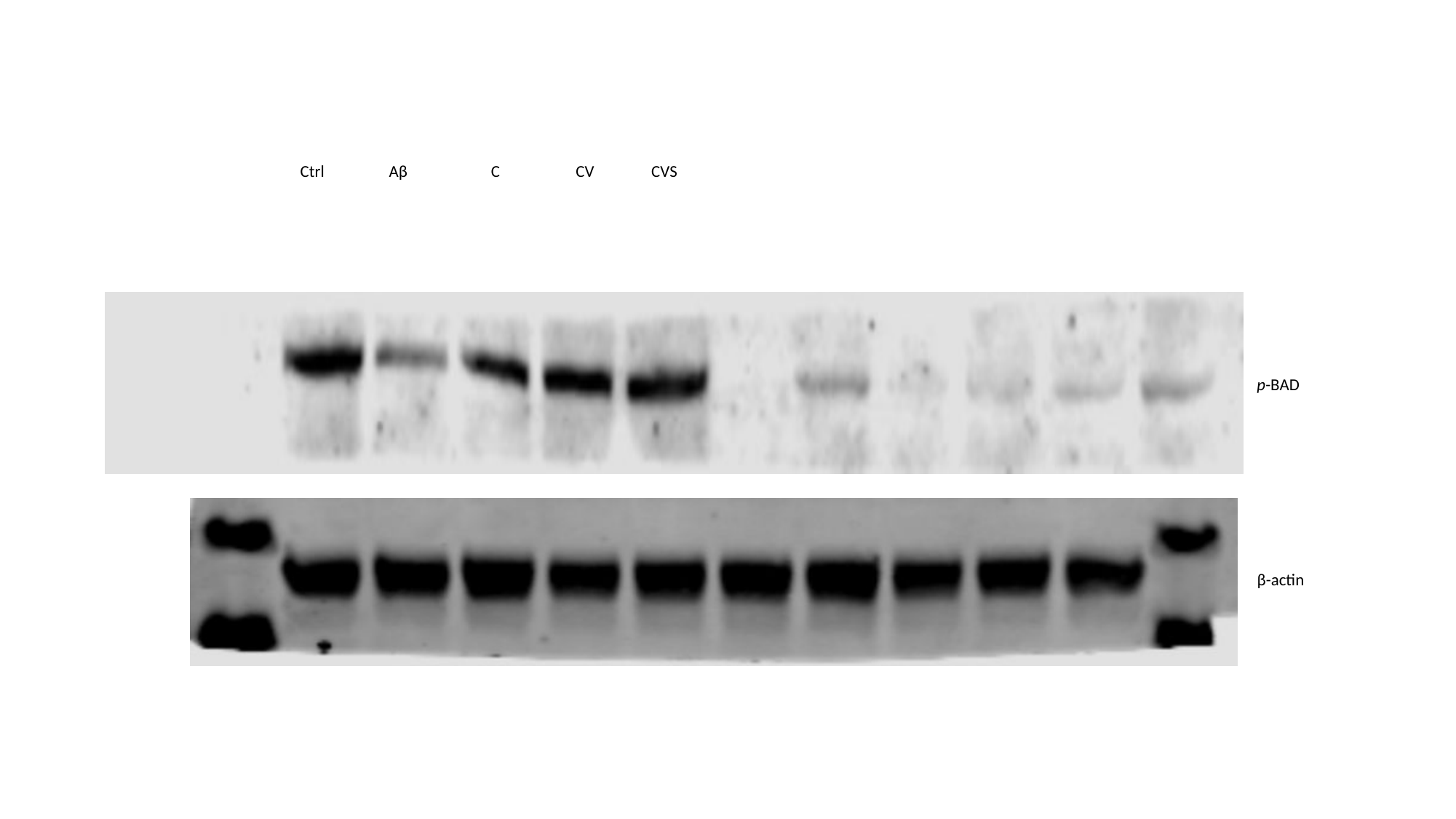

Ctrl Aβ C CV CVS
p-BAD
β-actin

Supplement: Supplemental Information 1 [file peerj-07-6716-s001.zip › Figure 3B.pptx]

## Slide 1
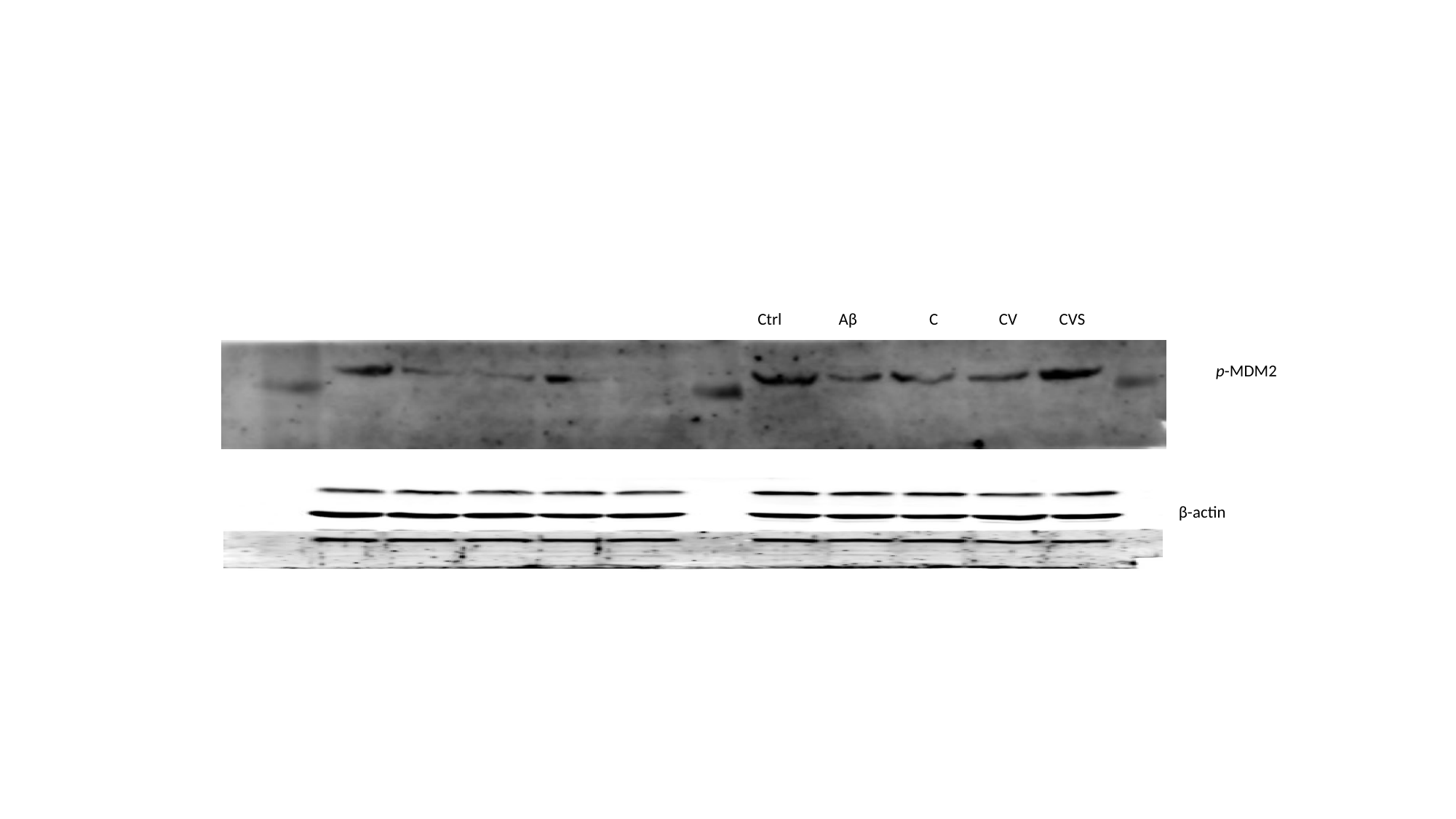

Ctrl Aβ C CV CVS
p-MDM2
β-actin

Supplement: Supplemental Information 1 [file peerj-07-6716-s001.zip › Figure 3C.pptx]

## Slide 1
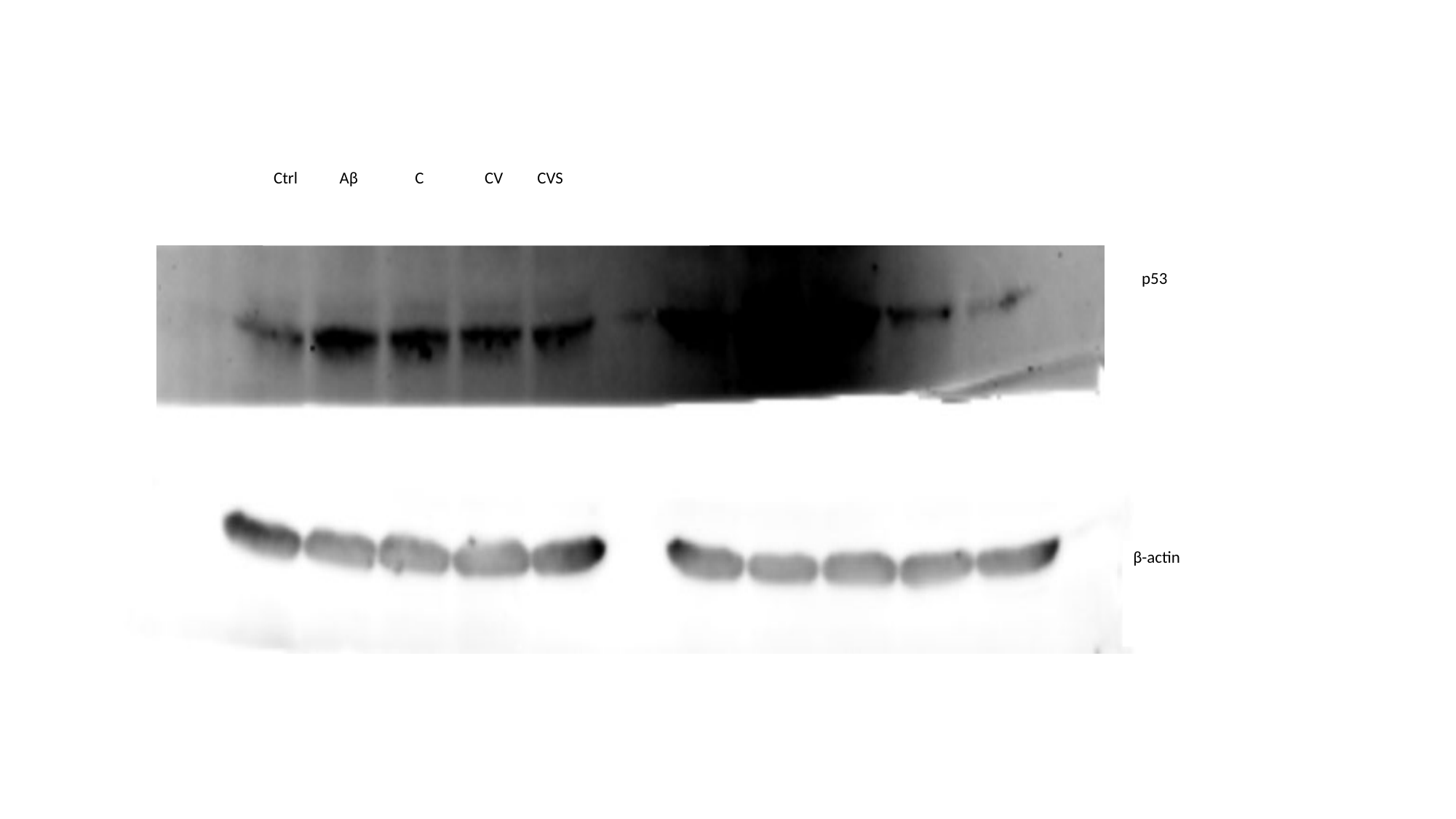

Ctrl Aβ C CV CVS
p53
β-actin

Supplement: Supplemental Information 1 [file peerj-07-6716-s001.zip › Figure 3D.pptx]

## Slide 1
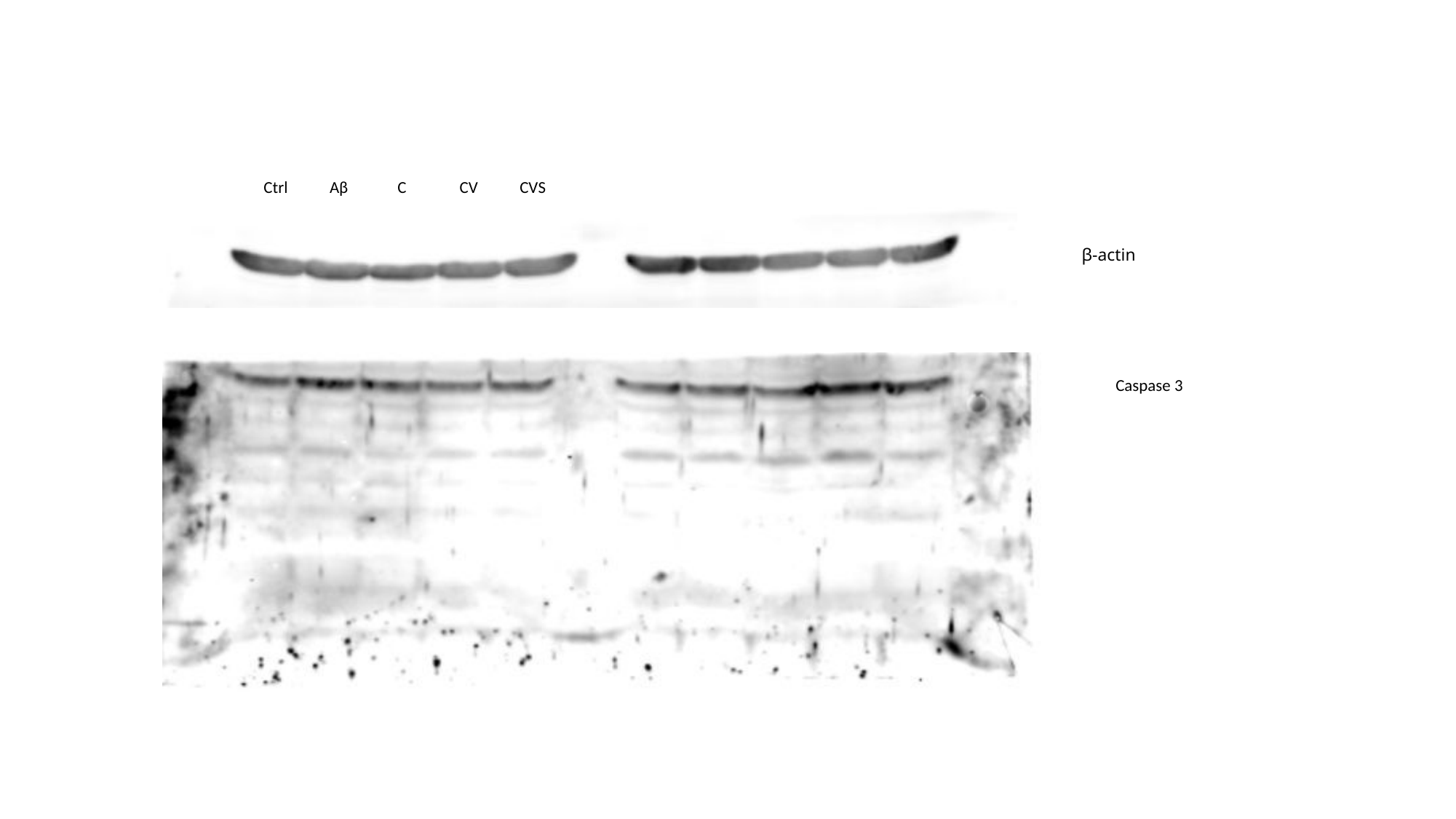

Ctrl Aβ C CV CVS
β-actin
Caspase 3

Supplement: Supplemental Information 1 [file peerj-07-6716-s001.zip › Figure 1C.pptx]
